# The vulnerability of hip fracture patients with cognitive impairment: an analysis of health conditions, hospital care, and outcomes

**DOI:** 10.1186/s12877-025-05744-9

**Published:** 2025-02-14

**Authors:** Dorothea Birkner, Mareen Pigorsch, Dorothee Riedlinger, Martin Möckel, Tobias Lindner, Liane Schenk, Johannes Deutschbein

**Affiliations:** 1 Charité—Universitätsmedizin Berlin, Corporate member of Freie Universität Berlin and Humboldt-Universität zu Berlin, Institute of Medical Sociology and Rehabilitation Science, Berlin, Berlin Germany; 2 Charité—Universitätsmedizin Berlin, Corporate member of Freie Universität Berlin and Humboldt-Universität zu Berlin, Institute of Biometry and Clinical Epidemiology, Berlin, Berlin Germany; 3 Charité—Universitätsmedizin Berlin, Corporate member of Freie Universität Berlin and Humboldt-Universität zu Berlin, Division of Emergency Medicine Campus Mitte and Virchow, Berlin, Berlin Germany

**Keywords:** Cognition, Cognitive impairment, Dementia, Vulnerable populations, Emergency department, Complications, Mortality, Hip fracture, Ageing, Elderly, Demographic change, Health services research

## Abstract

**Background:**

Cognitive impairment, including dementia, and hip fracture are both common among older patients. Both conditions are associated with increased morbidity and mortality. Cognitive impairment is often underdiagnosed and may remain undetected in hip fracture patients. Little is known about the prevalence, specific characteristics, and outcomes of hip fracture patients with cognitive impairment. This analysis aimed to compare hip fracture patients with and without cognitive impairments regarding their health conditions, hospital care, and the risk of complications and mortality.

**Methods:**

This study used data derived from the EMAAge project, a prospective multi-center cohort study conducted in Berlin, Germany. Patients aged 40 years and older with hip fracture were stratified into three cognitive status groups: no cognitive impairment (NCI), moderate cognitive impairment (MCI), and severe cognitive impairment (SCI). Categorization was based on patients’ ability to engage in interviews and their performance on the 6-item Cognitive Impairment Test (6-CIT). Standardized mean differences were used to compare various health-related parameters and health care utilization measures. Regression models, both adjusted and unadjusted, were calculated for the number of complications and the mortality rate.

**Results:**

Cognitive impairment was present in 37% of the 310 hip fracture patients in the study cohort. Patients with cognitive impairment had a worse baseline health profile, delayed admission to the emergency department, a longer time to surgery, and were less likely to be referred to a rehabilitation program. In the adjusted regression model for the number of complications, the incidence rate ratio was 1.237 (*p* = 0.292) for MCI patients and 2.065 (*p* < 0.001) for SCI patients compared with NCI patients. The adjusted odds ratio for mortality was 1.046 (*p* = 0.942) for MCI patients and 2.875 (*p* = 0.060) for SCI patients.

**Conclusions:**

Hip fracture patients with cognitive impairment, particularly severe impairment, arrive at the ED in a considerably poorer state of health and are at a higher risk of adverse outcomes, including complications and mortality. Timely identification of this at-risk group upon arrival appears to be essential to providing adequate care. This study highlights the need for interventions and research aimed at improving prevention, emergency care and outcomes for this vulnerable group, addressing their specific risk factors, and promoting the quality of care in hospital and after discharge.

**Supplementary Information:**

The online version contains supplementary material available at 10.1186/s12877-025-05744-9.

## Introduction

 As life expectancy continues to increase globally, we will experience an elderly demographic shift [1] and a growth in the number of cognitively impaired patients [2]. About 1.8 million people in Germany have been diagnosed with dementia or Alzheimer’s disease, and this number is expected to double in about 40 years [3, 4]. Worldwide, the number of dementia patients is expected to increase to 67.5 million by 2030, and by 2050, forecasts estimate around 131.5 million patients with dementia [5–7]. However, more than 50% of patients with dementia have not been formally diagnosed with the condition [8, 9]. A recent review estimated that around 37.0–50.0% of surgical patients had unrecognized cognitive impairment [10]. Patients and even caregivers benefit from early diagnosis and treatment of cognitive impairments because they are associated with various risks, e.g. accelerated cognitive decline and a faster reduction in quality of life [11]. Dementia, as the main manifestation of cognitive impairment in old age, is an independent risk factor for suffering a hip fracture, one of the most common and serious injuries in older people [12]. 

Cognitive impairment includes a spectrum ranging from subjective cognitive decline, mild cognitive impairment (MCI) to dementia, with MCI often considered an intermediate stage between normal cognitive aging and dementia [13, 14]. MCI can be defined as a cognitive state situated between normal cognition and dementia, while functional abilities are largely maintained. Dementia is typically characterized by the fact that the cognitive impairment has become severe enough to impair the person’s daily functioning [15]. MCI is often considered an intermediate stage between normal cognitive aging and dementia [16]. 

In 2019, 1.4 million cases of hip fractures were captured worldwide [17] and this number is predicted to rise to up to 4.5 million by the year 2050 [1, 18]. Total health care costs per patient, including the initial stay plus health and social care costs in the first year after a hip fracture, exceed $50,000 [19]. Femoral neck fractures were the most frequent fracture in Germany between 2009 and 2019, with an incidence of 120/100,000 citizens. Pertrochanteric fractures showed the second-highest incidence, with 109/100,000 citizens. The incidence of all hip fracture types increased by between 23% and 38% in this period of 10 years, explained by the demographic changes [20]. 

As only 40–60% of individuals suffering from a hip fracture regain the level of mobility they had before the fracture and 10–20% of affected individuals need to be institutionalized, hip fractures are a health hazard for elderly patients, negatively influencing their independence [1]. Approximately 22% of patients die within the year following a hip fracture [21, 22]. 

The proportion of cognitively impaired patients among hip fracture cases is high: estimates go up to 42% [23]. However, cognitive status is often not systematically assessed in clinical care. Hence, the specific needs and increased vulnerability of cognitively impaired hip fracture patients may remain unaddressed. Overall, there is insufficient data on the prevalence of cognitive impairments in hip fracture patients, their health condition, hospital care, and outcomes. To improve hospital and post-discharge care for these patients, a better understanding of their situation compared with non-impaired patients is needed [24]. 

The aim of our study was to examine the risk of complications and mortality in hip fracture patients with and without cognitive impairment. To achieve this, we investigated differences in patient characteristics, medical care, healthcare delivery, and health-related outcomes among patients with severe, moderate, and no cognitive impairment who sustained a hip fracture.

## Methods

### Study design

The EMAAge study is an observational, prospective multi-center cohort study of adult patients admitted to a hospital due to a hip fracture [25]. Conducted as part of the Emergency and Acute Medicine Network for Health Care Research in Berlin (EMANet) [26], the study received approval from the local ethics committee of Charité-Universitätsmedizin Berlin (EA1/362/16) and registered in the German Clinical Trials Register (DRKS00014273). Written consent was obtained from all participants.

### Setting

The EMAAge study was conducted from 2017 to 2019 in six inner-city emergency departments (EDs) in Berlin and included patients who sought ED treatment because of a hip fracture. The hospitals represented different levels of care, including two university hospitals. All clinical records of patients approaching the ED with suspected hip fracture were screened by a team of study nurses in the ED of the respective study site. After confirmation of the diagnosis, they approached patients within seven days after ED presentation during the in-hospital stay for information about and inclusion in the study. Following the acquisition of informed consent, participants were interviewed using a tablet-based, standardized questionnaire. This comprised instruments for different patient-reported outcomes and questions on patients’ health status and health care situation. A follow-up interview was conducted six months after discharge via telephone or as a paper-and-pencil questionnaire via mail.

The study questionnaires were available in German, English, Arabic, and Turkish.

In addition, the study entailed the collection of clinical data pertaining to the initial hospitalization from hospital information systems (HIS) using a standardized electronic case report form (eCRF). Further details of the study design and recruitment within the EMAAge study have been described previously [27]. 

### Participants & recruitment

All adult patients admitted to one of the six participating emergency departments (EDs) with a diagnosis of hip fracture, as defined by the International Classification of Diseases (IC) codes S72.0, S72.1, and S72.2 were eligible for inclusion in the study. Individuals who were critically ill or who lacked the requisite language skills in German, English, Turkish, and Arabic were excluded from participation in the study. In cases where patients lacked the requisite cognitive abilities to engage in the interview process independently due to dementia, relatives or legal guardians were invited to participate on the patients’ behalf. This decision was informed by prior diagnoses of dementia and, if no diagnosis was documented, by the assessment of clinical personnel at the respected wards. In alignment with the World Health Organization (WHO) Global Status Report on the Public Health Response to Dementia, the present analysis was conducted on all participants in the EMAAge study who were aged 40 years or older [28]. 

Of the 1.024 screened patients, 510 were eligible, and 344 patients consented to study participation. Reasons for ineligibility were a diagnosis other than the study diagnosis, critical illness, or language barriers. Comparison of our cohort with an unbiased sample of hip fracture patients, based on HIS data, showed no differences in gender or age. However, there are indications that the burden of comorbidities is slightly higher in our cohort compared to the general hip fracture population. Of the 510 patients who met the eligibility criteria, 166 declined to participate in the study [29]. 

### Variables, measurements, definitions, and outcomes

For the purposes of this analysis, the participants were classified into three distinct groups. The participants were divided into three groups based on their cognitive status: those with no cognitive impairment (NCI, group 1), those with mild cognitive impairment (MCI, group 2), and those with severe cognitive impairment (SCI, group 3). Cognitive impairment was defined in accordance with the test results of the 6-item cognitive impairment test (6-CIT), a validated dementia screening test suitable for use in a variety of clinical settings [30]. The test includes items on orientation, attention, memory, and calculations. The scoring system ranges from 0 to 28 points. Scores of 0–7 indicated normal cognitive function, scores from 8 indicate at least mild cognitive impairment. Patients unable to participate in the interview and the cognitive test due to a pre-existing dementia (proxy interviews) were categorized as severely cognitively impaired (SCI).

#### Outcomes

The outcomes of this study can be summarized as follows: (a) Mortality, defined as all-cause mortality occurring within six months of study enrollment; and (b) The number of complications experienced during the index hospital stay for hip fracture treatment. All documented post-surgical complications mentioned in the medical chart were recorded and categorized according to Carpintero et al. [31]. The following events and conditions were defined as complications: anemia, urinary tract infections, delirium, cardiac or pulmonary complications, electrolytic and metabolic disorders, and sepsis, or systemic inflammatory response syndrome (SIRS). The number of complications was calculated for each patient.

#### Measures and variables

Sociodemographic variables included age at presentation to the ED and sex. *Level of education* was recorded using the CASMIN classification and grouped into high, intermediate and basic education [32]. The *living situation* included the categories ‘living independently alone’, ‘living with other people’, and ‘living in a facility’ (nursing home).

#### Health parameters and hospital care

Fracture types were classified as femoral neck fracture, pertrochanteric fracture, subtrochanteric fracture and periprosthetic fracture. The interval between fall and presentation to the ED was self-reported in the following categories: “on the day of hospital admission”, “on the day before hospital admission” or “more than one day before admission”. Duration of time-to-surgery was determined by the interval between admission to the ED and the start of surgery. Surgical procedures were classified as: no surgery, implantation of total hip arthroplasty (THA), implantation of hemiprosthetic hip replacement (HHR), dynamic hip screw (DHS), proximal femoral nail (PFN), and screw osteosynthesis (SO). THA and HHA were categorized under arthroplasty, while DHS, PFN, and SO were classified as internal fixation. Length of stay (LOS) in the intensive care unit (ICU) was documented in days, in case of several ICU stays, the LOS consisted of all days spent in ICU. Duration of the overall hospital stay was captured in days.

#### Laboratory parameters

Laboratory parameters were collected from the first laboratory test at ED admission, including C-reactive protein (CRP, normal values < 5 mg/l; pathological values > 5 mg/L), leukocytes (normal values < 10.5/nL, pathological values > 10.5/nL), sodium (normal values 135–145 mmol/L, pathological values < 135 and > 145 mmol/L), hemoglobin (normal values for men 13.5–17 g/dL and for women 12–16 g/dL, pathological values for men < 13.0 g/dL and women < 12 g/dL) and creatinine (normal values for men < 1.1 g/dL and women < 0.9 g/dL, pathological values for men > 1.1 g/dL and women > 0.9 g/dL). Using the formula as in Levey et al. [33], glomerular filtration rate (GFR; normal values ≥ 90 ml/min, mildly reduced kidney function 60–89 ml/min, moderately to severely reduced kidney function to kidney failure < 60 ml/min) was calculated based on age, sex, and creatinine level.

#### Baseline health characteristics

The burden of comorbidities was characterized according to the Charlson Comorbidity Index (CCI) and based on all comorbidities documented in the medical record, including ICD-Code, and free text documentation [34]. Information on long-term care dependency as defined by German law was collected, representing the patients’ dependence on caregivers and ability to manage daily life.

*Malnutrition* was evaluated using the Short Nutritional Assessment Questionnaire (SNAQ) [35]. 

*Life satisfaction* was documented by asking patients to rate their overall satisfaction in life on a Likert Scale from “not at all satisfied”, scored as 0, to “completely satisfied”, scored as 10 [36]. The subjective level of *social support* was measured by the number of people one could rely on (none; 1–2; 3–5; more than 5).

#### Follow-up parameters

In the follow-up survey, patients were asked about their current *care level* and *life satisfaction*. In addition, information on health care utilization, such as *physical therapy* and *occupational therapy*, *hospital re-admissions*, and *ED re-visits* within the 6 months after surgery were collected.

All variables are presented in Supplement Table 1.

### Data analysis

In the first step of the analyses, health and health care parameters were analyzed, comparing patients with regard to their cognitive status (three groups). For categorical variables, we calculated absolute frequencies and proportions, and for metric variables, means and standard deviations. To evaluate the sizes of differences between the three cognition groups, standardized mean differences (SMD) were calculated. For each parameter, the average SMD of all three SMDs is reported, which allows for easy comparison of the various parameters.

To elucidate the association between cognitive status and the two outcomes in greater detail, a multivariable logistic regression was conducted for the outcome of mortality and a multivariable negative binomial regression was performed for the number of complications. According to the findings of previous studies, the regression model on complications was adjusted for age, gender, leukocyte levels, hyponatremia, GFR, pre-fracture care dependency, CCI, and malnutrition [37]. The model of mortality was adjusted for age, gender, CCI, malnutrition, pre-fracture care dependency, post-surgical complications (number of complications: none, one, two, or more complications), and whether patients were directly referred to a rehabilitation facility [38]. To control for center effects, the dichotomous variable “study center” (university hospital (2 sites) and general hospital (4 sites)) was included in both models. In addition, we calculated an unadjusted version for both regression models.

Multiple imputation were used to handle missing values within the regression models. The imputation models consisted of the variables in the corresponding analysis model and the CASMIN variable in addition. For the imputation of the malnutrition variable, we first imputed the three SNAQ variables and did a passive imputation. For both imputation models, we used the R package mice with m = 20 imputations [39]. The application of imputation permitted the calculation of multiple models, including those comprising patients for whom no data pertaining to cognition were available.

The analyses in this paper are explorative and not confirmatory. Therefore, correction for multiple testing was not performed. Due to the exploratory character of the models, we did not apply a certain p-value level to assume variables were worth considering as relevant for the outcomes. Descriptive statistics were performed using the statistics program IBM SPSS Statistics (version 27). The models were done using R version 4.1.1.

## Results

### Participants

The sample comprised of 323 patients aged 40 years and older, of whom 310 patients had valid information on cognitive status. Of this sample, 66.8% (*n* = 207) were female and 33.2% (*n* = 103) were male. The mean age was 76 years (SD 11.49). No signs of cognitive impairment were detected in 196 (63.2%) participants, while 50 (16.1%) participants were identified as cognitively impaired during the initial screening process. An additional 64 (20.7%) participants were unable to take part in the initial interview due to severe cognitive impairments and were included through a proxy (close relative or legal guardian). A total of *n* = 210 patients and proxies participated in the follow-up interview 6 months after hospital admission.

### Descriptives

Tables 1 and 2 present the characteristics of all individuals and the differences between the three cognition groups. In terms of the average standardized mean differences, cognition status showed a strong association with dependency on long-term care both pre-fracture (SMD = 1.235) and at follow-up (SMD = 1.221). Moderate effects were seen in the age of the patients, educational level (SMD = 0.469), living situation (SMD = 0.753) the type of hip fracture (SMD = 0.442), the burden of comorbidities (CCI: SMD = 0.678), the occurrence of delirium (SMD = 0.521), and the number of complications (SMD = 0.533), and subjective social support (SMD = 0.682). The admission laboratory values of leukocytes (SMD = 0.366) and CRP (SMD = 0.328), urinary tract infection (SMD = 0.349), ICU treatment (SMD = 0.373), Life satisfaction (at baseline: SMD = 0.493; at follow-up: SMD = 0.373), and mortality (SMD = 0.489) showed considerable differences. No relevant difference was seen in length of surgery (SMD = 0.047), length of stay (SMD = 0.089), gender distribution (SMD = 0.136), the sodium level at admission (SMD = 0.184), and the frequency of physical therapy after discharge (SMD = 0.165). In almost all parameters, SCI had worse characteristics than MCI and NCI: they arrived with greater delay in the ED, had worse laboratory values, more comorbidities, more complications, and a higher 6-month mortality rate. In two parameters, SCI scored better than MCI and NCI: they received more occupational therapy after discharge and had fewer hospital readmissions. In most parameters, the MCI group showed worse results than the NCI group and better results than the SCI group. They had the highest chances to receive rehabilitation directly after discharge, whereas their utilization of physical therapy in the community was lower than in both other groups.

**Table 1:** Baseline characteristics of total sample and according to cognition status

	**All patients at baseline** ***n* =310**	**No Cognitive Impairment (NCI)** ***n*=196**	**Moderate Cognitive Impairment (MCI)** ***n*=50**	**Severe Cognitive Impairment (SCI)** ***n* =64**	**Average Standardized mean difference**
	**n (%)**	**n (%)**	**n (%)**	**n (%)**	**(SMD)**^D^
**Sociodemographics**					
Age, mean (SD)	76.12 (11.49)	73.70 (11.87)	78.06 (9.17)	82.03 (9.45)	0.538
Sex^A^					0.136
Male	103 (33.2)	68 (34.7)	20 (40.0)	15 (23.4)	
Female	207 (66.8)	128 (65.3)	30 (60.0)	49 (76,6)	
Educational Status (CASMIN Classification)^B^					0.469
Basic	149 (49.2)	76 (39.2)	29 (61.7)	44 (71.0)	
Intermediate	91 (30.0)	66 (34.0)	12 (25.5)	13 (21.0)	
High	63 (20.6)	52 (26.8)	6 (12.8)	5 (8.1)	
Living situation					0.753
Living with others	108 (35.0)	78 (40.0)	18 (36.0)	12 (18.8)	
Living alone	148 (47.9)	104 (53.3)	25 (50.0)	19 (29.7)	
Living in a facility	53 (17.2)	13 ( 6.7)	7 (14.0)	33 (51.6)	
**Health parameters and hospital care**					
Fracture type					0.442
Femoral neck fracture	141 (45.5)	103 (52.6)	14 (28.0)	24 (37.5)	
Pertrochanteric fracture	145 (46.8)	74 (37.8)	34 (68.0)	37 (57.8)	
Subtrochanteric fracture	19 (6.1)	14 (7.1)	2 (4.0)	3 (4.7)	
Periprosthetic fracture	5 (1.6)	5 (2.6)	0 (0)	0 (0)	
Time of fall					0.293
Same day as presentation in ED	247 (79.9)	160 (81.6)	42 (84.0)	45 (71.4)	
On the day before presentation in ED	28 (9.1)	16 (8.2)	3 (6.0)	9 (14.3)	
Several days before presentation in ED	27 (8.7)	18 (9.2)	3 (6.0)	6 (9.5)	
Unknown	7 (2.2)	2 (1.0)	2 (4.0)	3 (4.8)	
**Laboratory values**					
C-reactive protein (CRP)^B^ >5 mg/l	93 (41.4)	47 (34.6)	15 (40.5)	31 (58.5)	0.328
Leukocytes^C^ >10.5/nl	139 (45.6)	74 (38.3)	25 (50.0)	40 (64.5)	0.366
Sodium^B^ <135 mmol/l	38 (13.2)	19 (10.9)	6 (12.0)	13 (20.6)	
Hemoglobin: ♂: <13.0 g/dl; ♀ <12.0 g/dl	126 (41.4)	72 (37.7)	19 (38.0)	35 (55.6)	0.243
Glomerular Filtration Rate ml/min (GFR)					
≥90	95 (30.6)	71 (36.2)	10 (20.0)	15 (23.4)	0.286
60-89	106 (34.2)	66 (33.7)	19 (38.0)	20 (31.3)	
<60	109 (35.2)	59 (30.1)	21 (42.0)	29 (45.3)	
**Health parameters at baseline**					
Charlson Comorbidity Index (CCI)					0.678
0	87 (28.1)	75 (38.3)	9 (18.0)	3 (4.7)	
1	69 (22.3)	33 (16.8)	16 (32.0)	20 (31.2)	
2	53 (17.1)	37 (18.9)	5 (10.0)	11 (17.2)	
>3	101 (32.6)	5 (26.0)	20 (40.0)	30 (46.9)	
Signs of malnutrition (SNAQ)	72 (24.4)	34 (17.5)	15 (31.9)	23 (42.6)	0.376
Care dependency at baseline, yes	116 (38.9)	42 (21.9)	18 (39.1)	56 (91.8)	1.235
Life satisfaction at Baseline, mean (SD)	6.74 (SD 2.68)	7.24 (SD 2.40)	6.48 (SD 2.74)	5.22 (SD 3.0)	0.493
Social support: persons to rely on:					0.682
None	14 (4.6)	6 (3.1)	4 (8.3)	4 (6.5)	
1-2	109 (35.7)	57 (29.2)	16 (33.3)	36 (58.1)	
3-5	113 (37.0)	73 (37.4)	23 (47.9)	17 (27.4)	
More than 5	66 (21.6)	58 (29.7)	5 (10.4)	3 ( 4.8)	
**Parameters of hospital stay**					
Time to surgery, in minutes, mean (SD)	1140 (SD 1521)	1025 (SD 950)	910 (SD 631)	1659 (SD 2784)	0.273
Surgical procedure					0.277
No surgery	6 (1.9)	3 (1.5)	1 (2.0)	2 (3.1)	
Arthroplasty	111 (35.5)	77 (39.3)	11 (22.0)	23 (35.9)	
Internal Fixation	196 (62.6)	116 (59.2)	38 (76.0)	39 (60.9)	
Length of surgery, in minutes, mean (SD)	78.77 (SD 38.48)	79.54 (SD 39.83)	76.90 (SD 33.59)	77.90 (SD 38.39)	0.047
Complications, yes	163 (52.6)	83 (42.3)	29 (58.0)	51 (79.7)	0.542
Number of complications, mean (SD)	1.17 (SD 1.71)	0.81 (SD 1.43)	1.24 (SD 1.44)	2.23 (SD2.21)	0.533
Occurrence of specific complications after surgery, yes					
Cardiac or pulmonary	33 (10.6)	15 (7.7)	6 (12.0)	12 (18.8)	0.222
Urinary tract infection	69 (22.3)	32 (16.3)	12 (24.0)	25 (39.1)	0.349
Anemia	81 (26.1)	42 (21.4)	15 (30.0)	24 (37.5)	0.238
Delirium	47 (15.2)	16 (8.2)	6 (12.0)	25 (39.1)	0.521
Admission to intensive care unit (ICU), yes	110 (34.2)	62 (28.4)	19 (38.0)	29 (53.7)	0.373
LOS ICU, in days, mean (SD)	3.22 (SD 5.12)	2.25 (SD 4.10)	3.00 (SD 4.63)	4.85 (SD 6.40)	0.330
LOS in days, mean (SD)	11.65 (SD 10.12)	11.22 (SD 8.96)	12.19 (SD 9.40)	12.56 (SD 13.54)	0.085
Direct referral to rehabilitation	190 (63.3)	115 (61.5)	39 (78.0)	36 (57.1)	0.304
Mortality within 6 months after surgery	40 (12.9)	13 (6.6)	5 (10.0)	22 (34.4)	0.489

^A^The variable sex did not record any patients with diverse sex

^B^Variables with more than 5% missing data (CASMIN: 5.5%, C-reactive Protein: 27.0%, Sodium: 7.1%)

^C^There were no patients with leukopenia, defined as a leukocyte laboratory value below 3.8/nl, at admission to ED

^D^The mean value of all three SMDs between all cognition groups

**Table 2:** Characteristics of total follow-up sample and according to cognition status

	All patients *n* = 210^A^	No Cognitive Impairment (NCI) *n* = 141	Moderate Cognitive Impairment (MCI) *n* = 30	Severe Cognitive Impairment (SCI) *n* = 39	Average Standardized mean difference
	**n (%)**	**n (%)**	**n (%)**	**n (%)**	**(SMD)**^B^
Care dependency, yes	100 49.3	48 35.0	13 48.1	39 100	1.221
Life satisfaction, mean (SD)	6.61 (SD 2.68)	7.24 (SD 2.40)	6.48 (SD 2.74)	5.64 (SD 2.52)	0.373
Physical therapy within 6 months after surgery, yes	152 (74.9)	105 (76.6)	19 (65.5)	28 (75.7)	0.165
Occupational therapy within 6 months after surgery, yes	31 (15.8)	16 (12.1)	4 (14.3)	11 (30.6)	0.308
Presentation in ED within 6 months after surgery, yes	37 (22.8)	24 (20.7)	4 (20.0)	9 (34.6)	0.222
Hospitalization within 6 months after surgery, yes	51 (24.8)	37 (26.8)	6 (20.7)	8 (20.5)	0.099

^A^The follow-up interview was completed by 67.7% of the initial cohort, 12.9% of the participants had deceased and 19.4% were lost to follow-up

^B^The mean value of all three SMDs between all cognition groups

### Outcomes

The number of complications was strongly associated with cognitive status, especially SCI, even when adjusting for other important risk factors. In the negative binomial regression model for the number of complications, the incidence rate ratio for SCI was twice as high as for NCI (IRR = 2.065; *p* < 0.001). For MCI, the IRR was 1.52 (*p* = 0.046) in the unadjusted model and 1.237 (*p* = 0.292) in the adjusted model. In addition, age, comorbidities, and hyponatremia considerably increased the likelihood of complications (Table 3). Mortality was strongly associated with SCI (unadjusted OR = 7.597; *p* < 0.001) but not with MCI (unadjusted OR = 1.58; *p* = 0.408). The inclusion of potential confounders reduced the effect strongly: the adjusted OR for SCI were only 2.875 (*p* = 0.060). The most important confounders were age (aOR = 1.048; *p* = 0.042), signs of malnutrition (aOR = 2.351; *p* = 0.036), the occurrence of complications (one complication: aOR = 3.189; *p* = 0.036; two or more complications: aOR = 3.356; *p* = 0.046), and the direct referral to rehabilitation (aOR = 2.375; *p* = 0.052) (Table 4).

**Table 3:** Negative binomial regression model for the number of complications in patients with hip fractures^g^

	IRR	Lower 95% CI	Upper 95% CI	*p*-value
**Unadjusted model**				
Intercept	0.805	0.659	0.983	0.034
Moderate cognitive impairment^c^	1.520	1.009	2.290	0.046
Severe cognitive impairment^c^	2.762	1.957	3.898	< 0.001
***Adjusted model***				
Intercept	0.103	0.032	0.326	< 0.001
Age	1.015	1.000	1.031	0.055
Gender (male)^a^	1.307	0.958	1.782	0.092
Elevated leukocyte levels	0.983	0.736	1.312	0.906
Hyponatremia	1.588	1.138	2.215	0.007
GFR 60–89^b^	1.252	0.841	1.864	0.270
GFR < 60^b^	1.494	0.984	2.269	0.060
Moderate cognitive impairment^c^	1.237	0.833	1.835	0.292
Severe cognitive impairment^c^	2.065	1.389	3.070	< 0.001
Pre-fracture care dependency	0.946	0.650	1.376	0.770
CCI (= 1)^d^	1.336	0.817	2.186	0.249
CCI (= 2)^d^	1.460	0.892	2.388	0.133
CCI (≥ 3)^d^	2.561	1.653	3.967	< 0.001
Malnutrition^e^	1.104	0.803	1.520	0.542
Study center (general hospital)^f^	1.057	0.784	1.426	0.717

*N* = 323; Reference categories: ^a^women, ^b^GFR > 90, ^c^no cognitive impairment, ^d^CCI = 0, ^e^no signs of malnutrition, ^f^university hospital

^g^Regression model using multiple imputation to handle missing values. Variables with missing values: elevated leucozyte levels = 7; cognitive impairment = 13; pre-fracture care dependency = 11; malnutrition = 21; direct referral to rehabilitation = 10; total number of missing values = 41

**Table 4:** Logistic regression model of 6-month mortality among patients with hip fractures^g^

	OR	Lower 95% CI	Upper 95% CI	*p*-value
**Unadjusted Model**				
Intercept	0.067	0.038	0.118	< 0.001
Moderate cognitive impairment^b^	1.580	0.536	4.659	0.408
Severe cognitive impairment^b^	7.597	3.545	16.281	0.000
***Adjusted Model***				
Intercept	0.000	0.000	0.015	< 0.001
Age	1.048	1.002	1.097	0.042
Gender (male)^a^	0.983	0.418	2.316	0.969
Moderate cognitive impairment^b^	1.046	0.315	3.472	0.942
Severe cognitive impairment^b^	2.875	0.959	8.616	0.060
Pre-fracture care dependency	1.110	0.392	3.145	0.845
CCI (= 1)^c^	1.361	0.295	6.287	0.693
CCI (= 2)^c^	1.013	0.198	5.189	0.988
CCI (≥ 3)^c^	3.117	0.783	12.406	0.108
Malnutrition^d^	2.351	1.062	5.205	0.036
Complications *n* = 1^e^	3.189	1.027	9.899	0.046
Complications *n* ≥ 2^e^	3.356	1.106	10.182	0.033
Direct referral to rehabilitation	2.375	0.995	5.671	0.052
Study center (general hospital)^f^	0.445	0.175	1.127	0.089

*N* = 323; Reference categories: ^a^women, ^b^no cognitive impairment, ^c^CCI = 0, ^d^no signs of malnutrition, ^e^no complications, ^f^university hospital

^g^Regression model using multiple imputation to handle missing values. Variables with missing values: cognitive impairment = 13, pre-fracture care dependency = 11, malnutrition = 21, direct referral to rehabilitation = 10, total number of missing values = 44

## Discussion

The aim of this study was to investigate the differences in patient characteristics, medical care, healthcare delivery, and the risk of complications and mortality among patients with severe, moderate, and no cognitive impairment who sustained a hip fracture. In our study, 16% (*n* = 50) of patients with hip fracture showed MCI, while 21% (*n* = 64) had SCI. In total, 37% displayed cognitive impairment, which is in line with comparable studies that reported 25 to 40% of hip fracture patients to be affected with cognitive impairment [40–43]. We found differences in almost all descriptive parameters. The most notable differences were observed between the SCI group and the other two groups (NCI and MCI), particularly with regard to the burden of comorbidities, pre-fracture care dependency, living situation, subjective social support, age, and education. Differences between NCI and MCI tended to be small.

When comparing our study to others, it is notable that many studies frequently examine patients with dementia while simultaneously excluding patients with other cognitive impairments. In only a few studies, the Mini-Mental State Examination was utilized post-surgery [44, 45], which is comparable to our grouping in terms of the gradation of cognitive impairment severity [46]. In most previous studies, no distinction was made between different levels of cognitive impairment.

When experiencing a fall, patients with moderate to severe cognitive impairment more frequently had a pertrochanteric fracture, which is a sign of increased fragility [47], while patients without cognitive impairment were more likely to have a femoral neck fracture.

The time of the fall was often further in the past for patients with SCI, indicating a delayed discovery after the fall or a delayed response from the patient’s caregivers. It is possible that the higher number of complications was also affected by delayed admission to the ED. This delay, which was observed in SCI, might be traced back to the limited ability of SCI to express themselves and the potentially dysfunctional communication between patients and caregivers. Indirectly, this delayed admission may have contributed to the increased mortality rate in SCI.

In the case of hip fractures, prompt intervention is crucial. A short time window between injury and surgery is associated with a lower risk of death and lower rates of postoperative pneumonia and pressure sores among elderly patients with hip fracture [48, 49]. 

Upon arrival in the ED, all laboratory values captured in our study indicate that patients with cognitive impairment exhibit worse baseline health conditions, with the worst values in SCI partially associated with higher mortality rates after surgery. They also showed risk signs for adverse events, such as abnormal blood values and higher inflammation values (CRP). Low blood hemoglobin [50–54] and severe renal impairment at admission are associated with higher mortality after hip fracture surgery. Some laboratory parameter deviations can be explained by advanced age, such as low GFR and low sodium values [55]. 

Moreover, the poor acute health state of patients with cognitive impairment was reflected in the burden of comorbidities. It is known that the degree of cognitive impairment in older adults with hip fractures may influence the presence of comorbidities and the potential complications that may arise [56]. 

Malnutrition is a condition in old age that is associated with frailty, poorer outcomes, and increased mortality in hip fracture patients [57]. There were more malnourished patients in the cohorts with cognitive impairment, which may be explained by reduced capacity for self-care or reduced appetite [58]. Corresponding to our population, comorbidities, acute diseases, medication, and cognitive disorders may also be accountable for the malnutrition of patients [59]. In Germany, rehabilitation programs are usually planned for three to four weeks. In addition to orthopedic rehabilitation, which is designed to restore mobility, there is a distinct program for older patients, known as geriatric rehabilitation, which aims to enhance patients’ autonomy across a range of activities. Studies on hip fracture rehabilitation have shown that it is crucial to prioritize convenient nutritional intake in patients with cognitive disorders to preserve their functional abilities [60] and prevent the progression of cognitive decline [61]. 

However, our data showed that SCI had the lowest proportion of direct referrals to rehabilitation. This may be attributed to considerations by the referring clinical staff. In order for a patient to participate in a rehabilitation program, it is necessary for the patient to demonstrate a presumed capacity for rehabilitation and to possess the requisite abilities to engage with the program. Future studies should examine clinical decision-making processes involving patients with cognitive impairment and ways to increase their participation in rehabilitation.

Cognitive impairment is a significant risk factor for long-term care dependency [62, 63] which was reflected in the study cohort as approximately 90% of patients with SCI were already dependent on nursing care at baseline, compared to just under 40% of MCI patients, and around 20% of patients with NCI. In the follow-up period, the same trend persisted, with approximately 10% more patients requiring care in all three groups.

Life satisfaction decreased with increasing cognitive impairment and continued to decline in the follow-up period. It is known that most patients experience a decrease in life satisfaction following a hip fracture, as they often do not regain their pre-fall mobility. Psychosocial factors, including life satisfaction, have a significant protective effect on hip fracture risk [64]. 

In addition to subjective assessments, other variables that contributed to improved outcomes were measurable factors, such as the time to surgery and the duration of the surgical procedure. The time from admission to surgery was found to be considerably prolonged for patients with SCI, slightly shorter for those with MCI, and the shortest for patients with NCI. There may be several reasons for this. The medical and organizational admission of patients with cognitive impairment can be complicated and prolonged by communication difficulties. As these patients have an increased number of medications and comorbidities, more time is needed to assess potential obstacles to surgery. In previous studies, it was observed that patients with cardiopulmonary conditions were only admitted for hip surgery after the accompanying symptoms had been clarified, resulting in a temporal delay between admission and the surgical procedure [65]. 

As is already established, a time-to-surgery exceeding 39–48 h is associated with an elevated mortality rate in patients with acute hip fractures [49, 66]. It is therefore imperative to identify vulnerable groups in the ED and to implement more expedient treatment regimens for this population, with the ultimate goal of reducing overall mortality.

A positive development emerged after the start of our study: In 2019, a new quality guideline for the treatment of patients with hip fracture was published by the German Joint Federal Committee (G-BA) [67]. This included the systematic screening of older patients in the ED to detect certain risk factors such as cognitive impairment and frailty.

The increased risk of complications in patients with cognitive impairment has been observed before, both in patients with hip fracture [5] and other fractures [64]. In Berggren et al.’s study [68], complications such as cardiac and pulmonary issues, urinary tract infections, and delirium were described with increased frequencies as well. However, anemia was not separately reported. The higher risk of anemia as a complication can be explained by the patient’s poorer condition, with already lower hemoglobin levels upon admission. Patients with hip fracture often arrive with anemia [69] and are already poorly prepared for their hip surgery, which often leads to additional blood loss. Postoperative anemia is an important risk factor that leads to frailty and weakness [70]. However, Barceló et al. demonstrated that patients would not benefit from pre-surgical blood transfusions [22]. Delirium is known to be more frequent in elderly patients with hip fracture and even more so if dementia has previously been diagnosed [71]. 

As cognitively impaired patients more often suffered from complications, SCI almost twice as often experienced treatment in an intensive care unit (ICU). Treatment in the ICU, however, is associated with an elevated risk of delirium. Consequently, complications and ICU treatment can be interdependent, particularly in patients with cognitive impairment [72, 73]. 

Complications are not only unpleasant but also have a significant impact on the risk of mortality, which is strongly impacted by cognitive status. This applied most of all to patients with severe cognitive impairments. A large proportion of the increased odds could be explained by other risk factors, namely age, malnutrition, comorbidities, perioperative complications, and the direct referral to a rehabilitation program. However, the effect in patients with severe cognitive impairment remained strong, even after adjustment. For moderate impairments, the effect disappeared when controlling for confounders. The association between cognitive impairment and mortality has been observed in several studies, both for short-term and long-term mortality [5, 38]. Most previous studies did not specifically look at moderately impaired patients, and there is a great heterogeneity regarding the adjustment for confounders. It seems remarkable that moderately impaired patients had the highest rate of direct rehabilitation. This might be interpreted as a sensitivity to the importance of post-surgery rehabilitation, especially for patients with cognitive impairment [74]. 

Following hospital discharge, SCI patients received enhanced treatment in one care parameter compared to the other groups: they received physical therapy more frequently, while patients with MCI received it less often than patients with NCI. This indicates that patients with pre-existing cognitive impairment received appropriate care following hip surgery, while patients whose cognitive impairment was only noticed during their hospital stay had less support. SCI patients appear to be well integrated into care structures that enable therapy, while patients with MCI might be less integrated and tend to be overwhelmed after hospital discharge. Occupational therapy was sought by more patients with cognitive impairment than patients with NCI in the 6 months after surgery, with a similarly increased number of SCI patients compared to MCI patients. To preserve functional abilities and prevent falling again, it is crucial to begin rehabilitation, including occupational therapy, immediately after surgery [75, 76]. 

Patients with SCI were more likely to present to the ED within six months of undergoing hip surgery than patients with MCI. However, patients with NCI were the least likely to present again. Around 23% of our study population presented in an ED. One previous study showed frequencies around 14% [77]. Rehospitalization occurred in about 25% of the study population, and more frequently in the group of patients with NCI. As SCI patients already benefit from a well-established daily support network, we proposed that their caregivers might admit their patients to the ED as a precaution when unsure about their health state. This could potentially increase the number of patients not qualifying for an in-hospital stay. Readmission to a hospital after 6 months has been examined previously, showing frequencies around 12% [78], 18% [77], 31% [79], and 32% [80].

## Strengths & limitations

In terms of our study’s strengths and limitations, it can be stated that we gathered real-world healthcare data through a multi-center, prospective study, with minimal exclusion criteria. This was made possible by conducting data collection in multiple languages.

Our approach to assessing and categorizing cognitive impairments was based on scientific and practical considerations: the categorization of severe cognitive impairment was not based on detailed medical history, or specific medication due to the problem of unrecognized impairments. Therefore, the inability to actively participate in the interview process and the presence of legal guardians seemed like a pragmatic solution. However, more specific information on medical history and medication could have helped to identify vulnerable patients in more detail. The 6-CIT, which was used to detect moderate cognitive impairments, is a well-established and validated screening instrument, comparable to the more often used MMST. Both instruments only provide a snapshot. There was a risk of confusing pre-existing cognitive impairments with post-surgical impairments such as delirium. However, when there were indications of delirium in the patient record, the interview was delayed until the condition had diminished.

The inclusion of cognitively impaired patients by proxy represents both a strength and a limitation. It is of great importance to include these most vulnerable patients in health research, and proxies may be the only feasible way to do this. However, it is inevitable that proxy responses will be biased to some degree, as they are unlikely to be identical with the responses that the patient would have given in person.

As a health services research project, the study relied on clinical data from the hospital information systems. Hence, clinical data were not collected to answer our research questions. Instead, they represent the routine procedures of hospital health services. With regard to complications, this can make it difficult to decide whether an anemia or a urinary tract infection occurred only after the surgery or had been present prior to the ED admission. However, when the patient record indicated pre-existing conditions such as low hemoglobin levels at admission, it was excluded from complications.

The mortality rate was determined from the state death register, ensuring the resulting data is highly accurate. Therefore, if patients relocated to barrier-free housing or a care facility and were challenging to reach without any indication of passing away, the lack of feedback was not considered indicative of death.

Finally, as an observational study with an exploratory purpose, our data are not meant to prove causal relations between cognitive status and the analyzed outcomes.

## Conclusions

Hip fracture patients with cognitive impairment are at a high risk of adverse outcomes. It is of great importance to identify cognitively impaired patients as soon as possible when they arrive at the ED. Their health condition is often worse than that of unimpaired patients, requiring further attention. Ongoing efforts to improve the in-hospital and follow-up care of older hip fracture patients need to be directed not only but especially to patients with cognitive impairment. In Germany, the new quality guideline for patients with hip fracture and its potential improvements need to be evaluated, with a special focus on cognitive impairment.

While many intervention trials exclude patients with cognitive impairment, more interventions and more research are needed that specifically address these vulnerable patients and their specific risk factors.

## Supplementary Information

Supplementary Material 1: Supplement Table 1. Description of analyzed variables.

## Data Availability

Due to data protection reasons, the raw data supporting the study results cannot be made publicly available. They can be accessed upon reasonable request and for scientific reasons only via the corresponding author.

## Abbreviations

aOR: Adjusted Odds Ratio
CASMIN: Comparative Analysis of Social Mobility in Industrial Nations
CCI: Charlson Comorbidity Index
CRP: C-reactive Protein
DHS: Dynamic hip screw
eCRF: Electronic Clinical Report Form
ED: Emergency department
EMAAge: Daily-life function and self-reported quality of life of geriatric patients before and after emergency treatment. Analysis of the status quo and potentials of health care improvements using the example of proximal femoral fracture
EMANet: Emergency and Acute Medicine Network for Health Care Research Berlin
G-BA: German Joint Federal Committee
GFR: Glomerular filtration rate
HHR: Hemiprosthetic hip replacement
HIS: Hospital Information System
ICD: International Statistical Classification of Diseases and Related Health Problems
ICU: Intensive Care Unit
ICU: Intensive Care Unit
LOS: Length of Stay
MCI: Patients with moderate cognitive impairment
MMST: Mini Mental State Test
NCI: Patients with no cognitive impairment
OR: Odds Ratio
PFN: Proximal Femur Nail
SCI: Patients with severe cognitive impairment
SD: Standard deviation
SIRS: Systemic Inflammatory Response Syndrome
SMD: Standardized mean difference
SNAQ: Short Nutritional Assessment Questionnaire
SO: Screw osteosynthesis
THA: Total hip arthroplasty
WHO: World Health Organization
6-CIT: Six-Item Cognitive Impairment Test

## References

[1] Dyer SM, Crotty M, Fairhall N, Magaziner J, Beaupre LA, Cameron ID, et al. A critical review of the long-term disability outcomes following hip fracture. BMC Geriatr. 2016;16(1):158. 10.1186/s12877-016-0332-0. 27590604 10.1186/s12877-016-0332-0 PMC5010762

[2] Bishop NA, Lu T, Yankner BA. Neural mechanisms of ageing and cognitive decline. Nature. 2010;464(7288):529–35. 10.1038/nature08983. 20336135 10.1038/nature08983 PMC2927852

[3] Michalowsky B, Kaczynski A, Hoffmann W. Ökonomische und gesellschaftliche Herausforderungen der Demenz in Deutschland – Eine Metaanalyse. Bundesgesundheitsblatt Gesundheitsforschung Gesundheitsschutz. 2019;62(8):981–92. 10.1007/s00103-019-02985-z. 31297549 10.1007/s00103-019-02985-z

[4] Blotenberg I, Hoffmann W, Thyrian JR. Dementia in Germany: epidemiology and prevention potential. Dtsch Arztebl Int. 2023;120:470–6. 10.3238/arztebl.m2023.0100. 37226316 10.3238/arztebl.m2023.0100 PMC10487668

[5] Hou M, Zhang Y, Chen AC, Liu T, Yang H, Zhu X, et al. The effects of dementia on the prognosis and mortality of hip fracture surgery: a systematic review and meta-analysis. Aging Clin Exp Res. 2021;33(12):3161–72. 10.1007/s40520-021-01864-5. 33913118 10.1007/s40520-021-01864-5

[6] Ali G, Guerchet M, Wu Y, Prince M, Prina M. World Alzheimer Report 2015: the global prevalence of dementia. London: AlzheImer’s DiseaseInternational; 2015. p. 10–27 https://www.alzint.org/resource/world-alzheimer-report-2015/.

[7] Prince M, Ali GC, Guerchet M, Prina AM, Albanese E, Wu YT. Recent global trends in the prevalence and incidence of dementia, and survival with dementia. Alzheimers Res Ther. 2016;8(1):23. 10.1186/s13195-016-0188-8. 27473681 10.1186/s13195-016-0188-8 PMC4967299

[8] Lang L, Clifford A, Wei L, Zhang D, Leung D, Augustine G, et al. Prevalence and determinants of undetected dementia in the community: a systematic literature review and a meta-analysis. BMJ Open. 2017;7(2): e011146. 10.1136/bmjopen-2016-011146. 28159845 10.1136/bmjopen-2016-011146 PMC5293981

[9] Iliffe S, Robinson L, Brayne C, Goodman C, Rait G, Manthorpe J, et al. Primary care and dementia: 1. Diagnosis, screening and disclosure. Int J Geriatr Psychiatry. 2009;24(9):895–901. 10.1002/gps.2204. 19226529 10.1002/gps.2204

[10] Kapoor P, Chen L, Saripella A, Waseem R, Nagappa M, Wong J, et al. Prevalence of preoperative cognitive impairment in older surgical patients.: a systematic review and meta-analysis. J Clin Anesth. 2022;76: 110574. 10.1016/j.jclinane.2021.110574. 34749047 10.1016/j.jclinane.2021.110574

[11] Bradford A, Kunik ME, Schulz P, Williams SP, Singh H. Missed and delayed diagnosis of dementia in primary care: prevalence and contributing factors. Alzheimer Dis Assoc Disord. 2009;23(4):306–14. 10.1097/WAD.0b013e3181a6bebc. 19568149 10.1097/WAD.0b013e3181a6bebc PMC2787842

[12] Wang HK, Hung CM, Lin SH, Tai YC, Lu K, Liliang PC, et al. Increased risk of hip fractures in patients with dementia: a nationwide population-based study. BMC Neurol. 2014;14:175. 10.1186/s12883-014-0175-2. 25213690 10.1186/s12883-014-0175-2 PMC4172891

[13] Roberts R, Knopman DS. Classification and epidemiology of MCI. Clin Geriatr Med. 2013;29(4):753–72. 10.1016/j.cger.2013.07.003. 24094295 10.1016/j.cger.2013.07.003 PMC3821397

[14] Smid J, Studart-Neto A, César-Freitas KG, Dourado MCN, Kochhann R, Barbosa B, et al. Subjective cognitive decline, mild cognitive impairment, and dementia - syndromic approach: recommendations of the Scientific Department of Cognitive Neurology and Aging of the Brazilian academy of Neurology. Dement Neuropsychol. 2022;16(3 Suppl 1):1–24. 10.1590/1980-5764-dn-2022-s101pt. 36533160 10.1590/1980-5764-DN-2022-S101PT PMC9745999

[15] Hugo J, Ganguli M. Dementia and cognitive impairment: epidemiology, diagnosis, and treatment. Clin Geriatr Med. 2014;30(3):421–42. 10.1016/j.cger.2014.04.001. 25037289 10.1016/j.cger.2014.04.001 PMC4104432

[16] Vega JN, Newhouse PA. Mild cognitive impairment: diagnosis, longitudinal course, and emerging treatments. Curr Psychiatry Rep. 2014;16(10):490. 10.1007/s11920-014-0490-8. 25160795 10.1007/s11920-014-0490-8 PMC4169219

[17] Global, regional, and national burden of bone fractures in 204 countries and territories, 1990–2019: a systematic analysis from the global burden of disease study 2019. Lancet Healthy Longev. 2021;2(9):e580-e92. 10.1016/s2666-7568(21)00172-0. 10.1016/S2666-7568(21)00172-0 PMC8547262 34723233

[18] Cooper C, Cole ZA, Holroyd CR, Earl SC, Harvey NC, Dennison EM, et al. Secular trends in the incidence of hip and other osteoporotic fractures. Osteoporos Int. 2011;22(5):1277–88. 10.1007/s00198-011-1601-6. 21461721 10.1007/s00198-011-1601-6 PMC3546313

[19] Williamson S, Landeiro F, McConnell T, Fulford-Smith L, Javaid MK, Judge A, et al. Costs of fragility hip fractures globally: a systematic review and meta-regression analysis. Osteoporos Int. 2017;28(10):2791–800. 10.1007/s00198-017-4153-6. 28748387 10.1007/s00198-017-4153-6

[20] Rupp M, Walter N, Pfeifer C, Lang S, Kerschbaum M, Krutsch W, et al. The incidence of fractures among the adult population of Germany–an analysis from 2009 through 2019. Dtsch Arztebl Int. 2021;118(40):665–9. 10.3238/arztebl.m2021.0238. 34140088 10.3238/arztebl.m2021.0238 PMC8727861

[21] Schnell S, Friedman SM, Mendelson DA, Bingham KW, Kates SL. The 1-year mortality of patients treated in a hip fracture program for elders. Geriatr Orthop Surg Rehabil. 2010;1(1):6–14. 10.1177/2151458510378105. 23569656 10.1177/2151458510378105 PMC3597289

[22] Barceló M, Torres OH, Mascaró J, Casademont J. Hip fracture and mortality: study of specific causes of death and risk factors. Archives Osteoporos. 2021;16(1):15. 10.1007/s11657-020-00873-7. 10.1007/s11657-020-00873-7 33452949

[23] Seitz DP, Adunuri N, Gill SS, Rochon PA. Prevalence of dementia and cognitive impairment among older adults with hip fractures. J Am Med Dir Assoc. 2011;12(8):556–64. 10.1016/j.jamda.2010.12.001. 21450227 10.1016/j.jamda.2010.12.001

[24] Gill N, Hammond S, Cross J, Smith T, Lambert N, Fox C. Optimising care for patients with cognitive impairment and dementia following hip fracture. Z Gerontol Geriatr. 2017;50(Suppl 2):39–43. 10.1007/s00391-017-1224-4. 28364260 10.1007/s00391-017-1224-4 PMC5408034

[25] Schneider A, Riedlinger D, Pigorsch M, Holzinger F, Deutschbein J, Keil T, et al. Self-reported health and life satisfaction in older emergency department patients: sociodemographic, disease-related and care-specific associated factors. BMC Public Health. 2021;21(1):1440. 10.1186/s12889-021-11439-8. 34289829 10.1186/s12889-021-11439-8 PMC8296655

[26] Schmiedhofer M, Inhoff T, Krobisch V, Schenk L, Rose M, Holzinger F, et al. [EMANet: a regional network for health services research in emergency and acute medicine]. Z Evid Fortbild Qual Gesundhwes. 2018:135–6. 10.1016/j.zefq.2018.07.009. 30122458 10.1016/j.zefq.2018.07.009

[27] Deutschbein J, Lindner T, Mockel M, Pigorsch M, Gilles G, Stockle U, et al. Health-related quality of life and associated factors after hip fracture. Results from a six-month prospective cohort study. PeerJ. 2023;11: e14671. 10.7717/peerj.14671. 36942001 10.7717/peerj.14671 PMC10024485

[28] World Health Organization. Global status report on the public health response to dementia. 2021. https://www.who.int/publications/i/item/9789240033245.

[29] Schneider A, Wagenknecht A, Sydow H, Riedlinger D, Holzinger F, Figura A, et al. Primary and secondary data in emergency medicine health services research – a comparative analysis in a regional research network on multimorbid patients. BMC Med Res Methodol. 2023;23(1):34. 10.1186/s12874-023-01855-2. 36739382 10.1186/s12874-023-01855-2 PMC9898937

[30] O’Sullivan D, O’Regan NA, Timmons S. Validity and reliability of the 6-item cognitive impairment test for screening cognitive impairment: a review. Dement Geriatr Cogn Disord. 2016;42(1–2):42–9. 10.1159/000448241. 27537241 10.1159/000448241

[31] Carpintero P, Caeiro JR, Carpintero R, Morales A, Silva S, Mesa M. Complications of hip fractures: a review. World J Orthop. 2014;5(4):402–11. 10.5312/wjo.v5.i4.402. 25232517 10.5312/wjo.v5.i4.402 PMC4133447

[32] Brauns H, Scherer S, Steinmann S. The CASMIN educational classification in international comparative research. In: Hoffmeyer-Zlotnik JHP, Wolf C, editors. Advances in cross-national comparison: a European working book for demographic and socio-economic variables. Boston: Springer US; 2003. p. 221–44. 10.1007/978-1-4419-9186-7_11.

[33] Levey AS, Stevens LA, Schmid CH, Zhang YL, Castro AF 3, Feldman HI, et al. A new equation to estimate glomerular filtration rate. Ann Intern Med. 2009;150(9):604–12. 10.7326/0003-4819-150-9-200905050-00006. 19414839 10.7326/0003-4819-150-9-200905050-00006 PMC2763564

[34] Roffman CE, Buchanan J, Allison GT. Charlson comorbidities index. J Physiother. 2016;62(3):171. 10.1016/j.jphys.2016.05.008. 27298055 10.1016/j.jphys.2016.05.008

[35] Kruizenga HM, Seidell JC, de Vet HCW, Wierdsma NJ, van Bokhorst–de MAE. Development and validation of a hospital screening tool for malnutrition: the short nutritional assessment questionnaire (SNAQ©). Clin Nutr. 2005;24(1):75–82. 10.1016/j.clnu.2004.07.015. 15681104 10.1016/j.clnu.2004.07.015

[36] Beierlein C, Kovaleva A, László Z, Kemper CJ, Rammstedt B. Eine Single-Item-Skala zur Erfassung der Allgemeinen Lebenszufriedenheit: Die Kurzskala Lebenszufriedenheit-1 (L-1). 2014. 10.6102/zis229.

[37] Visser A, Geboers B, Gouma DJ, Goslings JC, Ubbink DT. Predictors of surgical complications: a systematic review. Surgery. 2015;158(1):58–65. 10.1016/j.surg.2015.01.012. 25731783 10.1016/j.surg.2015.01.012

[38] Smith T, Pelpola K, Ball M, Ong A, Myint PK. Pre-operative indicators for mortality following hip fracture surgery: a systematic review and meta-analysis. Age Ageing. 2014;43(4):464–71. 10.1093/ageing/afu065. 24895018 10.1093/ageing/afu065

[39] van Buuren S, Groothuis-Oudshoorn K. Mice: multivariate imputation by chained equations in R. J Stat Softw. 2011;45(3):1–67. 10.18637/jss.v045.i03.

[40] Li L, Bennett-Brown K, Morgan C, Dattani R. Hip fractures. Br J Hosp Med (Lond). 2020;81(8):1–10. 10.12968/hmed.2020.0215. 32845763 10.12968/hmed.2020.0215

[41] Kristoffersen MH, Dybvik E, Steihaug OM, Bartz-Johannesen CA, Martinsen MI, Ranhoff AH, et al. Validation of orthopaedic surgeons’ assessment of cognitive function in patients with acute hip fracture. BMC Musculoskelet Disord. 2019;20(1):268. 10.1186/s12891-019-2633-x. 31153373 10.1186/s12891-019-2633-x PMC6545206

[42] Gruber-Baldini AL, Zimmerman S, Morrison RS, Grattan LM, Hebel JR, Dolan MM, et al. Cognitive impairment in hip fracture patients: timing of detection and longitudinal follow-up. J Am Geriatr Soc. 2003;51(9):1227–36. 10.1046/j.1532-5415.2003.51406.x. 12919234 10.1046/j.1532-5415.2003.51406.x

[43] Olofsson E, Gustafson Y, Mukka S, Tengman E, Lindgren L, Olofsson B. Association of depressive disorders and dementia with mortality among older people with hip fracture. BMC Geriatr. 2023;23(1):135. 10.1186/s12877-023-03862-w. 36890449 10.1186/s12877-023-03862-w PMC9996856

[44] Juliebø V, Krogseth M, Skovlund E, Engedal K, Ranhoff AH, Wyller TB. Delirium is not associated with mortality in elderly hip fracture patients. Dement Geriatr Cogn Disord. 2010;30(2):112–20. 10.1159/000318819. 20733304 10.1159/000318819

[45] Ruggiero C, Bonamassa L, Pelini L, Prioletta I, Cianferotti L, Metozzi A, et al. Early post-surgical cognitive dysfunction is a risk factor for mortality among hip fracture hospitalized older persons. Osteoporos Int. 2017;28(2):667–75. 10.1007/s00198-016-3784-3. 27717957 10.1007/s00198-016-3784-3

[46] Upadhyaya AK, Rajagopal M, Gale TM. The six item cognitive impairment test (6-CIT) as a screening test for dementia: comparison with Mini-mental State Examination (MMSE). Curr Aging Sci. 2010;3(2):138–42. 10.2174/1874609811003020138. 20158493 10.2174/1874609811003020138

[47] Abrahamsen B, Laursen HVB, Skjødt MK, Jensen MH, Vestergaard P. Age at hip fracture and life expectancy in Denmark - secular trends over two decades. Bone. 2020;130: 115083. 10.1016/j.bone.2019.115083. 31622776 10.1016/j.bone.2019.115083

[48] Simunovic N, Devereaux PJ, Sprague S, Guyatt GH, Schemitsch E, Debeer J, et al. Effect of early surgery after hip fracture on mortality and complications: systematic review and meta-analysis. CMAJ. 2010;182(15):1609–16. 10.1503/cmaj.092220. 20837683 10.1503/cmaj.092220 PMC2952007

[49] Polok K, Pilecki Z, Kutaj-Wąsikowska H, Kutryba B, Sunol R, Pilecki G, et al. Time from admission to surgery in Polish patients with hip fractures: temporal trends in the last decade and association with duration of hospitalization and in-hospital mortality. Pol Arch Intern Med. 2020;130(6):506–11. 10.20452/pamw.15342. 32380820 10.20452/pamw.15342

[50] Mosfeldt M, Pedersen OB, Riis T, Worm HO, Mark S, Jørgensen HL, et al. Value of routine blood tests for prediction of mortality risk in hip fracture patients. Acta Orthop. 2012;83(1):31–5. 10.3109/17453674.2011.652883. 22248167 10.3109/17453674.2011.652883 PMC3278654

[51] Haddad BI, Hamdan M, Alshrouf MA, Alzubi A, Khirsheh A, Al-Oleimat A, et al. Preoperative hemoglobin levels and mortality outcomes after hip fracture patients. BMC Surg. 2023;23(1):266. 10.1186/s12893-023-02174-5. 37658363 10.1186/s12893-023-02174-5 PMC10474652

[52] Praetorius K, Madsen CM, Abrahamsen B, Jørgensen HL, Lauritzen JB, Laulund AS. Low levels of Hemoglobin at Admission are Associated with increased 30-Day mortality in patients with hip fracture. Geriatr Orthop Surg Rehabil. 2016;7(3):115–20. 10.1177/2151458516647989. 27551568 10.1177/2151458516647989 PMC4976734

[53] Yombi JC, Putineanu DC, Cornu O, Lavand'homme P, Cornette P, Castanares-Zapatero D. Low haemoglobin at admission is associated with mortality after hip fractures in elderly patients. Bone Joint J. 2019;101-b(9):1122–8. 10.1302/0301-620x.101b9.Bjj-2019-0526.R1. 31474150 10.1302/0301-620X.101B9.BJJ-2019-0526.R1

[54] Sayed-Noor A, Al-Amiry B, Alwan A, Knutsson B, Barenius B. The association of on-admission blood hemoglobin, C-reactive protein, and serum creatinine with 2-year mortality of patients with femoral neck fractures. Geriatr Orthop Surg Rehabil. 2021;12: 21514593211037758. 10.1177/21514593211037758. 34422440 10.1177/21514593211037758 PMC8377304

[55] Soiza RL, Hoyle GE, Chua MP. Electrolyte and salt disturbances in older people: causes, management and implications. Reviews Clin Gerontol. 2008;18(2):143–58. 10.1017/S0959259809002822.

[56] Jiménez Mola S, Calvo-Lobo C, Idoate Gil J, Seco Calvo J. Cognitive impairment level and elderly hip fracture: implications in rehabilitation nursing. Rehabil Nurs. 2020;45(3):147–57. 10.1097/rnj.0000000000000159. 29985871 10.1097/rnj.0000000000000159

[57] Franz K, Deutschbein J, Riedlinger D, Pigorsch M, Schenk L, Lindner T, et al. Malnutrition is associated with six-month mortality in older patients admitted to the emergency department with hip fracture. Front Med (Lausanne). 2023;10: 1173528. 10.3389/fmed.2023.1173528. 37153099 10.3389/fmed.2023.1173528 PMC10158933

[58] Sanford AM, Morley JE, Berg-Weger M, Lundy J, Little MO, Leonard K, et al. High prevalence of geriatric syndromes in older adults. PLoS One. 2020;15(6): e0233857. 10.1371/journal.pone.0233857. 32502177 10.1371/journal.pone.0233857 PMC7274399

[59] Roberts HC. Changing the food environment: the effect of trained volunteers on mealtime care for older people in hospital. Proc Nutr Soc. 2018;77(2):95–9. 10.1017/s0029665117002804. 29081312 10.1017/S0029665117002804

[60] Wojzischke J, van Wijngaarden J, van den Berg C, Cetinyurek-Yavuz A, Diekmann R, Luiking Y, et al. Nutritional status and functionality in geriatric rehabilitation patients: a systematic review and meta-analysis. Eur Geriatr Med. 2020;11(2):195–207. 10.1007/s41999-020-00294-2. 32297199 10.1007/s41999-020-00294-2

[61] Nijmeijer WS, van Dartel D, de Groot R, Woudsma S, Folbert EC, den Braber N, et al. Transparency in hip fracture recovery over institutional boundaries: the transmural monitoring pathway. Clin Rehabil. 2023;37(10):1406–19. 10.1177/02692155231166120. 36991558 10.1177/02692155231166120

[62] Blüher S, Schilling R, Stein T, Gellert P. [Preventing the need for long-term care: analyses of assessment data from the medical service]. Bundesgesundheitsblatt Gesundheitsforschung Gesundheitsschutz. 2023;66(5):490–7. 10.1007/s00103-023-03685-5. 36944805 10.1007/s00103-023-03685-5 PMC10163063

[63] Seitz DP, Gill SS, Gruneir A, Austin PC, Anderson GM, Bell CM, et al. Effects of dementia on postoperative outcomes of older adults with hip fractures: a population-based study. J Am Med Dir Assoc. 2014;15(5):334–41. 10.1016/j.jamda.2013.12.011. 24524851 10.1016/j.jamda.2013.12.011

[64] Peel NM, McClure RJ, Hendrikz JK. Psychosocial factors associated with fall-related hip fractures. Age Ageing. 2007;36(2):145–51. 10.1093/ageing/afl167. 17259636 10.1093/ageing/afl167

[65] Tabu IA, Araneta KTS, Alpuerto BB 2nd, Delgado GD, Lai JGL, San Juan JAG, et al. Improving fragility hip fracture care through data: a multicentre experience from a country with an emerging economy during the COVID-19 pandemic. BMJ Open Qual. 2023;12(Suppl 2). 10.1136/bmjoq-2023-002299. 10.1136/bmjoq-2023-002299 PMC10565130 37783523

[66] Kristiansson J, Hagberg E, Nellgård B. The influence of time-to-surgery on mortality after a hip fracture. Acta Anaesthesiol Scand. 2020;64(3):347–53. 10.1111/aas.13494. 31652349 10.1111/aas.13494

[67] Richtlinie des Gemeinsamen Bundesausschusses über Maßnahmen zur Qualitätssicherung zur Versorgung von Patienten mit einer hüftgelenknahen Femurfraktur gemäß § 136 Absatz 1 Satz 1 Nummer 2 für nach § 108 SGB V zugelassene Krankenhäuser. 2023. https://www.g-ba.de/richtlinien/118/.

[68] Berggren M, Stenvall M, Englund U, Olofsson B, Gustafson Y. Co-morbidities, complications and causes of death among people with femoral neck fracture - a three-year follow-up study. BMC Geriatr. 2016;16:120. 10.1186/s12877-016-0291-5. 27260196 10.1186/s12877-016-0291-5 PMC4893237

[69] Nissenholtz A, Levy Y, Cooper L, Bugaevsky Y, Weiss A, Beloosesky Y. [Anemia in patients after hip fracture repair surgery]. Harefuah. 2020;159(9):689–93. 32955813

[70] Sheehan KJ, Williamson L, Alexander J, Filliter C, Sobolev B, Guy P, et al. Prognostic factors of functional outcome after hip fracture surgery: a systematic review. Age Ageing. 2018;47(5):661–70. 10.1093/ageing/afy057. 29668839 10.1093/ageing/afy057

[71] Lin X, Cao Y, Liu X, Yu K, Miao H, Li T. The hotspots and publication trends in postoperative delirium: a bibliometric analysis from 2000 to 2020. Front Aging Neurosci. 2022;14: 982154. 10.3389/fnagi.2022.982154. 36225889 10.3389/fnagi.2022.982154 PMC9549321

[72] Sieber FE, Mears S, Lee H, Gottschalk A. Postoperative opioid consumption and its relationship to cognitive function in older adults with hip fracture. J Am Geriatr Soc. 2011;59(12):2256–62. 10.1111/j.1532-5415.2011.03729.x. 22092232 10.1111/j.1532-5415.2011.03729.x PMC3245376

[73] Bramley P, McArthur K, Blayney A, McCullagh I. Risk factors for postoperative delirium: an umbrella review of systematic reviews. Int J Surg. 2021;93:106063. 10.1016/j.ijsu.2021.106063. 34411752 10.1016/j.ijsu.2021.106063

[74] Cadel L, Kuluski K, Wodchis WP, Thavorn K, Guilcher SJT. Rehabilitation interventions for persons with hip fracture and cognitive impairment: a scoping review. PLoS One. 2022;17(8):e0273038. 10.1371/journal.pone.0273038. 35969624 10.1371/journal.pone.0273038 PMC9377630

[75] McLaren AN, Lamantia MA, Callahan CM. Systematic review of non-pharmacologic interventions to delay functional decline in community-dwelling patients with dementia. Aging Ment Health. 2013;17(6):655–66. 10.1080/13607863.2013.781121. 23611141 10.1080/13607863.2013.781121 PMC3723698

[76] Mohr S, Müller C, Hildebrand F, Laubach M. Sturzprävention bei älteren Menschen durch ergotherapeutische Wohnraumanalyse, -beratung und -anpassung: eine Prozessdarstellung. Z Gerontolo Geriatrie. 2023;56(5):408–14. 10.1007/s00391-022-02103-w. 10.1007/s00391-022-02103-w PMC10406676 36070010

[77] Shyu YI, Liang J, Tseng MY, Li HJ, Wu CC, Cheng HS, et al. Enhanced interdisciplinary care improves self-care ability and decreases emergency department visits for older Taiwanese patients over 2 years after hip-fracture surgery: a randomised controlled trial. Int J Nurs Stud. 2016;56:54–62. 10.1016/j.ijnurstu.2015.12.005. 26742606 10.1016/j.ijnurstu.2015.12.005

[78] Sun L, Wang C, Zhang M, Li X, Zhao B. The surgical timing and prognoses of elderly patients with hip fractures: a retrospective analysis. Clin Interv Aging. 2023;18:891–9. 10.2147/cia.S408903. 37287528 10.2147/CIA.S408903 PMC10243344

[79] McGee AS, Johnson JL, Paul KD, Patel HA, Christie MC, Williamson BD, et al. Risk factors for readmission following revision total hip arthroplasty in patients undergoing surgery for noninfective causes. Cureus. 2018;10(7): e2934. 10.7759/cureus.2934. 30202666 10.7759/cureus.2934 PMC6128606

[80] Härstedt M, Rogmark C, Sutton R, Melander O, Fedorowski A. Impact of comorbidity on 6-month hospital readmission and mortality after hip fracture surgery. Injury. 2015;46(4):713–8. 10.1016/j.injury.2014.12.024. 25627481 10.1016/j.injury.2014.12.024

