# Supplementary material for: The vulnerability of hip fracture patients with cognitive impairment: an analysis of health conditions, hospital care, and outcomes
Source: BMC Geriatr. 2025 Feb 14;25:99. doi: 10.1186/s12877-025-05744-9 (PMC11829398; doi:10.1186/s12877-025-05744-9)
Supplement: Supplementary file 1 — Supplementary Material 1: Supplement Table 1. Description of analyzed variables. [file 12877_2025_5744_MOESM1_ESM.docx]

# Appendix

Supplement Table 1 Description of analyzed variables

| **Variable** | **Description** |
| --- | --- |
| Age | 40 years and above |
| Gender | Male/female |
| Level of education | According to CASMIN classification |
| Living situation | Living with others, living alone, living in a facility |
| Fracture type | Femoral neck, pertrochanteric, subtrochanteric or periprosthetic fracture |
| Comorbidities | Charlson Comorbidity Index |
| Care level at Baseline and Follow up | Level of care according to German social code XI (yes/no) |
| Life satisfaction at Baseline and Follow up | Ranging from 0 to 10 |
| Social support: Persons to rely on | None, 1-2, 3-5, mor than 5, I don’t know / NA |
| Laboratory value C-reactive protein | ≥ 0.3 mg/dL |
| Laboratory value Leukocytes | cells/nl |
| Laboratory value Hemoglobin | g/dl |
| Laboratory value Natrium | mmol/l |
| Glomerular filtration rate (GFR) | ml/min |
| Time of fall | Same day as, on the day before, several days before presentation in emergency department |
| Duration of admission to surgery | hh:mm |
| Surgical procedure | Hip total endoprosthesis (TEP), hip hemiendoprosthesis (HEP), dynamic hip screw (DHS), Proximal Femoral Nail (PFNA), Screw osteosynthesis (SO) |
| Duration of surgery | minutes |
| Duration of Intensive Care Unit | days |
| Duration of hospital stay | days |
| Physical therapy within 6 months after surgery | Yes/No |
| Occupational therapy within 6 months after surgery | Yes/No |
| Presentation in Emergency Department (ED) within 6 months after surgery | Yes/No |
| Hospitalization within 6 months after surgery | Yes/No |
| Death | Yes/No, date |
| Complications | Yes/No |
| Number of complications | n |
| Specific complication: Cardial or pulmonary | Yes/No |
| Specific complication: Urinary tract infection | Yes/No |
| Specific complication: Anemia | Yes/No |
| Specific complication: Delirium | Yes/No |
